# Sclerostin antibody promotes alveolar bone regeneration after tooth extraction

**DOI:** 10.17305/bb.2025.12999

**Published:** 2025-10-22

**Authors:** Erdal Ergünol, Rabia Şemsi, Duygu Dayanır, Remzi Orkun Akgün, Okan Ekim, Altay Uludamar, Ayhan Özkul, Aylin Sepici Dinçel

**Affiliations:** 1 Innovation, Education, Consultation and Organization Company, Alter Group, Istanbul, Türkiye; 2 Department of Medical Biochemistry, Institute of Health Sciences, University of Gazi, Ankara, Türkiye; 3 Department of Histology and Embryology, Faculty of Medicine, University of Gazi, Ankara, Türkiye; 4 Department of Anatomy, Faculty of Dentistry, University of Çankırı Karatekin, Çankırı, Türkiye; 5 Department of Anatomy, Faculty of Veterinary Science, University of Ankara, Ankara, Türkiye; 6 Department of Pathology, Faculty of Veterinary Science, Emeritus, University of Ankara, Ankara, Türkiye; 7 Department of Medical Biochemistry, Faculty of Medicine, University of Gazi, Ankara, Türkiye

**Keywords:** Sclerostin-ab, bone regeneration, graft material

## Abstract

Sclerostin is a key inhibitor of the Wnt signaling pathway, functioning by binding to the LRP5/6 receptor. This interaction inhibits beta-catenin expression, resulting in the downregulation of osteogenic markers, which contributes to the promotion of osteoporosis and an increase in osteoclast numbers. The primary objective of this research was to investigate the effects of sclerostin antibody (Scl-ab) on bone formation utilizing graft materials in tooth sockets, and to analyze the regulatory interaction between sclerostin and bone tissue through targeted sclerostin inhibition and stimulation of bone formation in tooth extraction sockets following local, single-dose administration. In this study, New Zealand male rabbits (3 months old, weighing 2.5–3 kg) were fully randomized to minimize bias. The experiments were conducted across five groups: a control group, a graft group, and three experimental groups receiving 100%, 75%, and 50% doses of Scl-ab. Calculated doses of Scl-ab were administered alongside the graft material in the extraction sockets, with results assessed at 2- and 4-week intervals. Cone-beam computed tomography indicated that the tooth extraction sockets treated with varying ratios of Scl-ab with graft material exhibited a statistically significant increase in the mean mandibular BV/TV ratio compared to the control and graft groups, with variations based on time and dosage. While bone volume improved over time, the most significant enhancement was observed in the 100% Scl-ab group. Additionally, the administration of different doses of Scl-ab significantly increased trabecular thickness (Tb.Th.) of the alveolar bone compared to both the control (*P* < 0.001) and graft (*P* < 0.001) groups, with histological analysis corroborating these findings. The therapeutic application of Scl-ab facilitates early bone formation, and the localized inhibition of sclerostin secreted within the bone microenvironment targets potential bone regeneration.

## Introduction

Extensive research in bone biochemistry has identified the Wnt/β-catenin pathway as a pivotal signaling cascade in skeletal physiology. This pathway is implicated in various cellular processes, including embryonic development, tissue homeostasis, and bone metabolism [1, 2]. Specifically, the Wnt/β-catenin pathway facilitates osteoblast differentiation, proliferation, and bone matrix synthesis, all of which are fundamental to bone formation. Its regulatory function in balancing bone resorption and formation during the remodeling cycle has established this pathway as a significant pharmacological target for skeletal disorders.

The Wnt/β-catenin pathway is tightly regulated by extracellular antagonists, such as sclerostin, a secreted glycoprotein predominantly produced by osteocytes. Acting as a negative regulator, sclerostin binds to the low-density lipoprotein receptor-related proteins (LRP5/6) co-receptors, leading to the phosphorylation and degradation of β-catenin, thus suppressing osteoblast maturation and new bone formation [3, 4]. Osteocytes, through the secretion of sclerostin, play a crucial role in signaling the termination of the remodeling phase. Structurally, sclerostin comprises 213 amino acids and has a molecular weight of 22–24 kDa. It belongs to the bone morphogenetic protein (BMP) antagonist family and is encoded on chromosome 17 within the 17q12-q21 region by the SOST gene [5–8].

Although sclerostin is most abundant in bone, it is also present in cartilage, kidney, liver, pancreas, heart, and plasma. While its precise functions in these tissues remain incompletely understood, it is believed to regulate the activity of bone-forming and bone-resorbing cells, thereby maintaining bone homeostasis [9, 10]. Sclerostin acts as a potent anti-anabolic factor, with its expression modulated by mechanical loading and cytokine signaling in bone tissue, ultimately influencing sclerostin levels in bones [8–10]. Winkler et al. first identified sclerostin in adult human osteocytes. Elevated sclerostin levels inhibit osteoblastogenesis, the formation of new bone tissue, and reduce overall bone formation, while downregulation of sclerostin expression enhances bone mass [1, 7, 11–13].

Targeting sclerostin with neutralizing antibodies has emerged as a promising therapeutic strategy for skeletal regeneration. Disrupting the interaction between LRP5/6 and sclerostin is essential for Wnt-related metabolic processes that impact bone health. Consequently, in our previous study, we targeted the loop 2 region of sclerostin, which binds stably to LRP5/6, and employed a series of *in silico* approaches, including molecular docking and molecular dynamics simulations, to screen drug-like compounds from the Drug-Bank database [14].

Recent investigations have focused on the systemic and subcutaneous administration of sclerostin antagonists. While these studies have provided valuable insights into its therapeutic potential, further research is necessary to fully elucidate its applications. A promising area of study is the use of sclerostin antagonists, such as romosozumab, blosozumab, and bps804, to promote bone formation and treat osteoporosis [15]. Additionally, the rare high bone mass disorder sclerosteosis and Van Buchem disease are associated with beta-catenin-dependent Wnt signaling linked to loss-of-function mutations in SOST. The cellular and molecular mechanisms underlying glucocorticoid-induced osteoporosis involve the suppression of Wnt signaling, resulting in inhibited osteoblast differentiation. Furthermore, during immobilization-induced osteoporosis, elevated sclerostin concentrations contribute to increased inhibition of bone formation.

Experimental studies have provided compelling evidence supporting sclerostin inhibition in bone repair. For example, Nakashima et al. (2024) demonstrated enhanced bone formation and wound healing in a tooth extraction socket using a bisphosphonate-induced osteonecrosis of the jaw (ONJ) model in conjunction with severe periodontitis to evaluate bone development in wild-type (WT) and sclerostin knockout mice [16]. Periodontitis causes permanent damage to soft tissue and alveolar bone, leading to tooth mobility and eventual tooth loss. This irreversible cascade of bone loss necessitates therapeutic intervention. A recent study by Nascimento et al. (2024) ranked periodontitis as the sixth-most prevalent health condition globally, and the Bangkok Declaration of 2024 emphasized “No Health Without Oral Health” [17].

The rationale for this study was to investigate the therapeutic potential of sclerostin inhibition for bone regeneration following tooth extraction. Specifically, we aimed to determine whether local, single-dose administration of a sclerostin-neutralizing antibody (Scl-ab), in combination with a bone graft, could enhance bone formation by reducing bone resorption. The primary objective was to evaluate the efficacy of targeted sclerostin inhibition in accelerating early socket healing and improving bone quality in a rabbit model.

## Materials and methods

### Experimental animal preparation, extraction of teeth, and treatment protocols

Male New Zealand albino rabbits, approximately three months old and weighing between 2.5 and 3 kg, were selected for this study to ensure physiological consistency. Prior to anesthesia, the rabbits were acclimated to their environment for several days to enhance their comfort. They were individually housed in cages equipped with environmental controls to maintain stable temperature and humidity, and they had continuous access to a standard diet and water. Following preliminary research, the extraction of the first premolars was determined to be the most effective approach. The rabbits were randomly assigned to three experimental groups and two control groups to minimize potential bias in the results.

Initially, all rabbits were anesthetized using xylazine HCl (5 mg/kg, intramuscularly) and ketamine (35 mg/kg, intramuscularly). A customized mouth opener (Utility Model Patent 2021–008511, “Auxiliary Oral Support for Veterinary Applications”) was then applied to maintain the mouth in an open position. A standardized experimental protocol for tooth extraction was implemented, as outlined by Ergünol et al. (2025) [18]. The right and left premolar teeth were extracted over two consecutive weeks, allowing for four weeks of healing for the right premolars and two weeks for the left.

Subsequent to the premolar tooth extraction, the alveolar sockets were treated according to the established protocol. The extraction sites were filled with graft material (Cerabone Granulate, grain size 0.5–1.0 mm, Straumann, Botis Biomaterials, Ref No: BO1515, GmbH) combined with sclerostin antibody (Scl-ab, abcam 63097). The socket entry was sutured, and the animals were sacrificed two weeks post-extraction. The groups included a control group (Group 1, tooth extraction only with physiological healing), a graft material group (Group 2, 50 mg graft material), and experimental groups (Group 3, Scl-ab 100%, 0.02 mg Scl-ab in 50 mg graft material; Group 4, Scl-ab 75%; and Group 5, Scl-ab 50%, each in 50 mg graft material).

### Radiologic analysis

**Figure 1.**
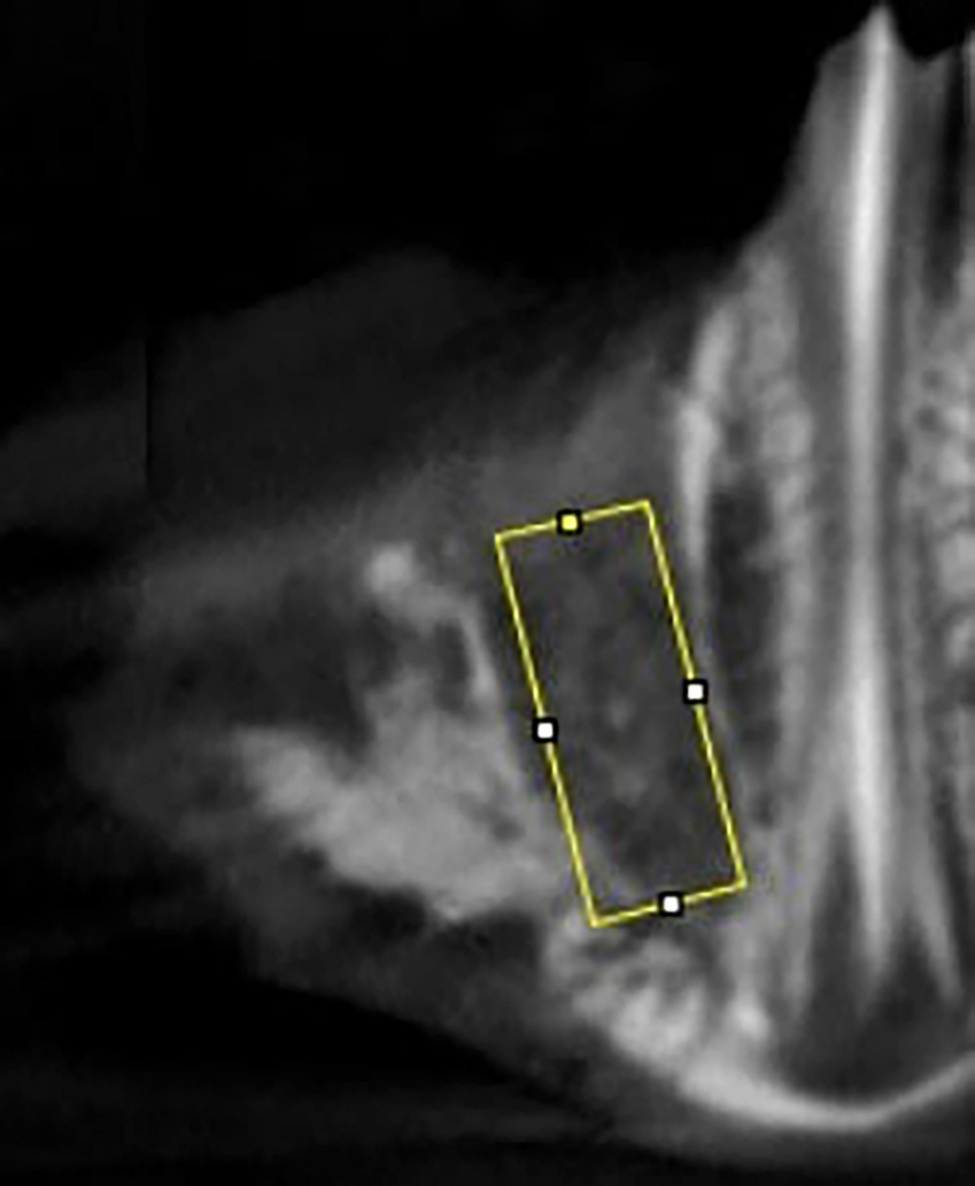
**Standardized CBCT region of interest (ROI) for alveolar bone quantification.** This parasagittal CBCT slice illustrates the mandibular premolar region. The ROI extends from the alveolar crest to the tooth apex, maintaining a consistent area of 24.63 mm^2^ across all specimens. This standardization facilitates the comparable extraction of BV/TV, Tb.Th., and Tb.Sp. Abbreviations: CBCT: Cone-beam computed tomography; ROI: Region of interest; BV/TV: Bone volume/total volume; Tb.Th.: Trabecular thickness; Tb.Sp.: Trabecular separation.

This study utilized the HDX WILL DENTRl 3D cone beam computed tomography (CBCT) device, manufactured by HDX WILL Corp. in Korea, for imaging. The CBCT device was configured to cover a specific field of view (FOV) measuring 160 × 80 mm. To ensure unbiased results, dose adjustments were made by a single technician. After irradiation, images were reconstructed using specific parameters: 80–100 kV, 4–10 mA, a voxel size of 200 µm, and an acquisition time of 8–24 s. Subsequently, a single operator analyzed the images on axial, sagittal, frontal, and cross-sectional sections using Cybermed On-demand software from Cybermed Inc. in Korea. Further image processing was conducted with CS_3D imaging software developed by Carestream Health Inc. in Rochester, NY, USA. Volumetric measurements were performed on a standardized region of interest, consisting of the alveolar bone surrounding the socket area of the premolar tooth. This region was defined as the area between the alveolar crest and the apex of the teeth, standardized to 24.63 mm^2^ for each specimen (Figure 1). A single investigator delineated the periphery of the desired alveolar bone region on 2D parasagittal images to maximize the measurement of bone formation while minimizing the inclusion of cavities. Bone volume relative to total volume, trabecular thickness (Tb.Th.), and trabecular separation (Tb.Sp.) were subsequently calculated for each specimen. An automated enclosing convex polygon method was implemented, with modifications to the Density distribution BoneJ ImageJ plug-in. This application included multiple steps to specify cortical results, mass and density distribution, and concentric density distribution [19–22].

### Histological analysis

The tissue samples from the entire mandible underwent a meticulous preparation process to ensure precision in analysis. Initially, they were immersed in a 10% neutral formaldehyde solution with a pH of 7.3 for 24 h to fix and preserve their structure. Decalcification was performed using EDTA at a pH of 7.4 to remove calcium deposits that could interfere with analysis [23]. Following fixation and decalcification, the tissues were thoroughly washed with water and subjected to washes in 70%, 80%, and 96% ethyl alcohol for 20 min each to eliminate any residual formaldehyde and EDTA. The tissues were then washed with acetone in four rounds, each lasting 20 min, to eradicate any remaining water and alcohol.

Subsequently, the tissues were submerged in Xylene I and Xylol II for 30 min each to eliminate traces of acetone. Once cleaned, the tissues were placed in Paraffin I and Paraffin II and melted at 60 ^∘^C for one hour, allowing for embedding in paraffin blocks. Sections of 6-µm thickness were produced using a microtome. The hematoxylin–eosin staining procedure was employed to examine the general histomorphology of the sections, which were then assessed under light microscopy. To ensure accuracy, each section was meticulously analyzed using an advanced PC-based image analysis system (Leica Qwin, Leica Ltd., Germany).

### Ethical statement

All animal experiments were conducted in accordance with the Guidelines for the Care and Use of Laboratory Animals, which were established, reviewed, and approved by the Local Ethics Committee for Animal Experiments at the University of Gazi (G.Ü.ET-20.007). The experimental procedures primarily adhered to the Animal Research: Reporting of *in vivo* Experiments (ARRIVE) guidelines, ensuring ethical, transparent, and reproducible animal research.

### Statistical analysis

All statistical analyses were performed using SPSS software for Windows, version 18.0 (SPSS Inc., Chicago, IL, USA). Descriptive statistics are reported as means ± standard deviations (SD), with a significance level set at 5% (*P* < 0.05) for all tests. The distribution of the data was evaluated using the Shapiro–Wilk test and visually confirmed with box plots. Levene’s test was utilized to examine the homogeneity of variances, thereby validating the parametric analyses. Subgroup analyses were conducted based on experimental weeks, comparing the control, graft, and sclerostin groups separately.

A mixed-design analysis of variance (mixed ANOVA) was employed to assess both within-group (time) and between-group effects on bone density. When significant main or interaction effects were identified, post hoc tests were applied for pairwise comparisons, with corrections for multiple comparisons to control the type I error rate. Post hoc results were reported with 95% confidence intervals (CIs), including both lower and upper bounds of the mean differences. If the assumptions for parametric testing were not met, non-parametric alternatives were utilized; specifically, Wilcoxon signed-rank tests were conducted for paired comparisons where appropriate. To ensure the robustness of the study design, a post-hoc power analysis was performed to evaluate whether the sample size provided sufficient statistical power (>80%) to detect meaningful differences across groups and time points. Effect sizes (Cohen’s d or partial eta squared, depending on the test) were also calculated to complement *P* values, quantifying the magnitude of observed effects.

## Results

This experimental research involved a comprehensive analysis of the effects of Scl-ab treatment on alveolar bone formation. We conducted an in-depth investigation to assess the inhibitory influence of sclerostin on bone formation during local administration. Utilizing CBCT scans, we measured various aspects of mandibular bone volume in a rabbit model following a single dose of Scl-ab combined with graft material over periods of 2 and 4 weeks.

Five rabbits were assigned to each group in the study. Given that sockets were obtained from the same rabbits at both time points, we acknowledged that the assumption of independent samples might be partially violated. Hence, the unit of analysis was defined as “animal,” rather than “socket,” and statistical analyses were performed using mean values per animal. A mixed-design ANOVA revealed significant differences between the groups (F(4,20) ═ 2.074.938, *P* < 0.0001). Effect size calculations indicated a highly significant effect size with η^2^ ═ 0.9999 and Cohen’s f ≈ 644.

A post-hoc power analysis, considering the observed variance and sample size, yielded a power of 1–β ═ 1.0. This finding indicates that the statistical power of the study is maximized, affirming that the observed differences are robust and not attributable to inadequate sampling.

**Figure 2.**
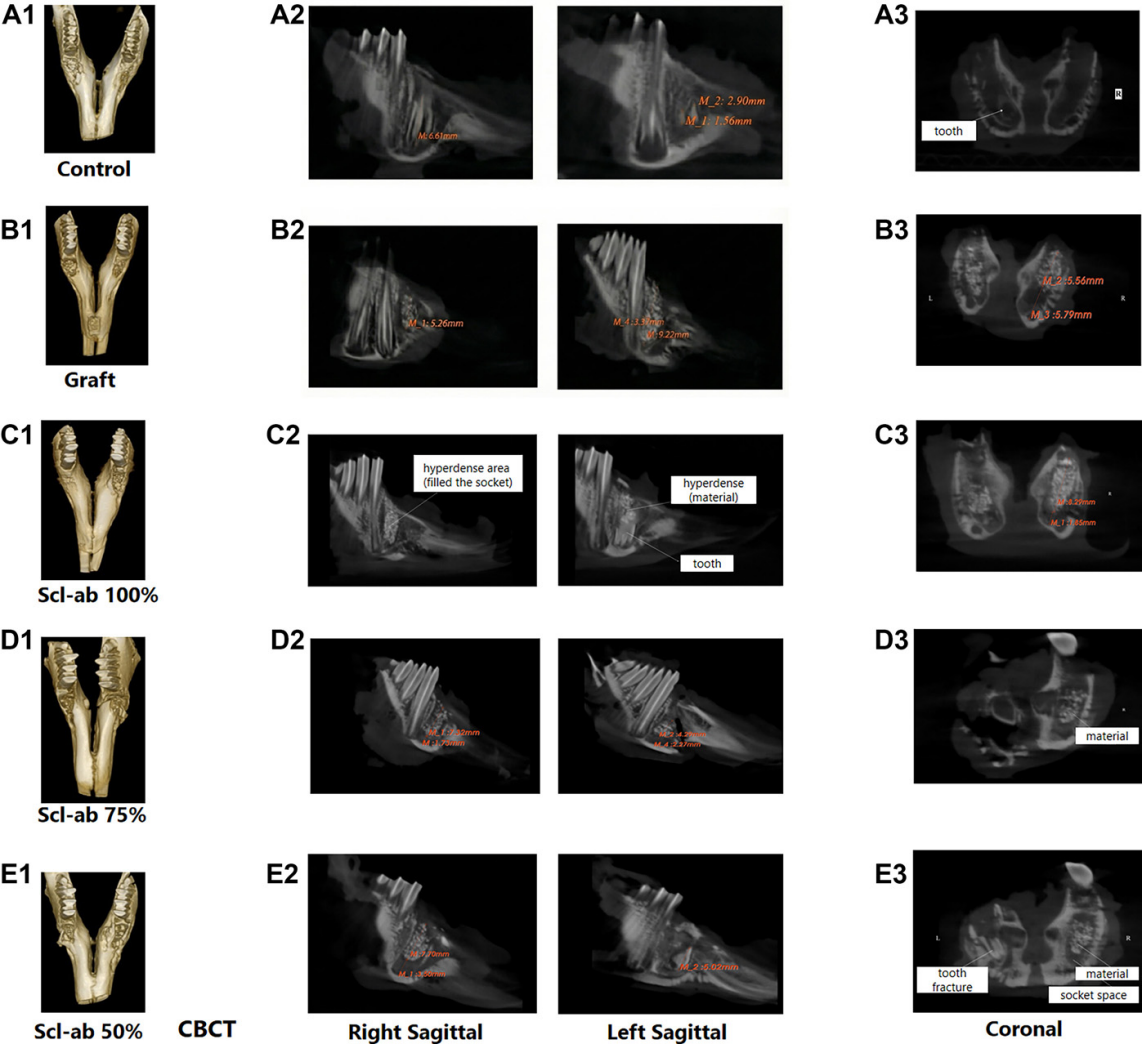
**Selected 3D CBCT images: right sagittal views (2 weeks) and left sagittal views (4 weeks of treatment) for all groups.** CBCT was utilized to capture 3D images, as well as sagittal (A2–E2) and coronal views (A3–E3), at 2- and 4-week intervals across various treatment groups (control, graft, and Scl-ab). These images illustrate the differences in bone regeneration within the defect area, highlighting the visual variations in the extent of bone formation among the treatment modalities. Abbreviations: CBCT: Cone-beam computed tomography; Scl-ab: Sclerostin antibody.

The selected three-dimensional images from the CBCT results are illustrated in Figure 2. The right sagittal views of all groups represent the 2-week treatment, while the left sagittal views depict the 4-week treatment.

Figure 2A1 illustrates the control group. After excluding the negligible tooth remnant, the empty socket area exhibited hypodensity with minimal bone regeneration. The empty socket areas showed no evidence of new bone formation, resulting in a predominantly unfilled defect. By the 4-week assessment, these trends persisted, with the alveolar space remaining hypodense and largely empty. Figure 2B1 illustrates the graft group. This image reveals the application of a grafting procedure to the first premolar on both sides. The graft group demonstrated significant advancements in bone healing by week 2. In the right coronal view, a hypodense area measuring 5.79 mm, representing the alveolar space, contrasts with a hyperdense area of 5.56 mm that corresponds to the graft material. The left coronal view indicates that the entire socket is filled with graft material and appears hyperdense. In the left sagittal view, a hyperdense region corresponding to the graft material measures 3.37 × 9.22 mm. By week 4, both the volume and density of the socket significantly increased. The left coronal view confirmed that the entire socket was filled with graft material, appearing hyperdense. Figure 2C1 displays the results for Scl-ab-filled tooth sockets on both sides of the first premolars. The right coronal view demonstrates an alveolar space of 1.85 mm and a solid region of 8.29 mm, nearly filling the socket. The right sagittal view reveals a dense area covering most of the socket. In the left coronal view, a dense area of approximately 6.27 mm is observed between two empty regions measuring 2.63 mm and 1.78 mm, respectively. The left socket contains less filling material compared to the right socket. Figure 2D1 presents the right sagittal image, where a dense material measuring approximately 7.32 mm in length is noted, nearly filling the socket. Additionally, a less dense alveolar space measuring 1.73 mm is observed. The left sagittal image reveals a dense area of 4.29 mm and a less dense area of 2.27 mm within the alveolar space. Figure 2E1 depicts the Scl-ab-filled socket with 50% of the total material, as illustrated in the right sagittal image, showing a hyperdense region measuring 7.7 mm and a slightly hypodense socket cavity of 3.5 mm. A hyperdense tooth fracture approximately 5.02 mm in length is observed in the left sagittal image. The coronal and axial images corroborate these findings, with no evidence of material flare observed.

Levene’s test results indicated that variance homogeneity was achieved between the groups in the 2-week measurements for the BV/TV parameter, with similar variances noted (F(4, 20) ═ 1.294, *P* ═ 0.306). However, variance distribution was not homogeneous at the 4-week mark, with significant differences observed between the groups (F(4, 20) ═ 16.668, *P* < 0.0001). Regarding the Tb.Th. parameter, variance homogeneity was not achieved for either the 2nd week (F(4, 20) ═ 2.992, *P* ═ 0.044) or the 4th week (F(4, 20) ═ 3.400, *P* ═ 0.028). When examining the Tb.Sp. measurements, the variances were homogeneous in the 2nd week (F(4, 20) ═ 1.635, *P* ═ 0.205), but the assumption of homogeneity was violated in the 4th week, revealing significant differences among the groups (F(4, 20) ═ 19.254, *P* < 0.0001). To enhance the manuscript, the primary endpoints were reassessed using robust methods. Robust tests (Welch’s ANOVA and Brown–Forsythe) were conducted to evaluate group differences for bone morphometric parameters, as the assumption of homogeneity of variances was not fully met. The analyses revealed statistically significant differences among groups for all variables except for the 4th week Tb.Sp. For BV/TV, Welch’s test indicated a significant group effect, F(4, 9.54) ═ 164.69, *P* < 0.001, which was confirmed by the Brown–Forsythe test, F(4, 12.40) ═ 130.77, *P* < 0.001. For 4-week BV/TV, Welch’s test yielded F(4, 9.01) ═ 596.03, *P* < 0.001, and Brown–Forsythe showed F(4, 9.52) ═ 135.60, *P* < 0.001. For 2-week Tb.Th., Welch’s test was significant, F(4, 9.23) ═ 64.35, *P* < 0.001, as was Brown–Forsythe, F(4, 13.73) ═ 48.08, *P* < 0.001. Similarly, for 4-week Tb.Th., Welch’s test showed F(4, 8.09) ═ 82.75, *P* < 0.001, and Brown–Forsythe yielded F(4, 12.66) ═ 54.56, *P* < 0.001. For 2-week Tb.Sp., Welch’s test was significant, F(4, 8.05) ═ 59.93, *P* < 0.001, and Brown–Forsythe confirmed this result, F(4, 14.10) ═ 30.68, *P* < 0.001. However, for 4-week Tb.Sp., robust tests could not be performed due to at least one group exhibiting zero variance, which precluded the computation of test statistics.

Additionally, the results of Mauchly’s sphericity test confirmed that the sphericity assumption was met for all parameters (BV/TV, Tb.Th., and Tb.Sp.) (*W* ═ 1.000, χ^2^=0.000, *P* ═ 1.000); therefore, no sphericity correction was necessary (Table 1).

Considering the within-subjects effects, the statistical analysis of the linear time effect indicated a significant impact on the BV/TV parameter (F(1, N) ═ 71.814, *P* < 0.001, partial η^2^ ═ 0.782). However, the interaction between time and group was not significant for the linear contrast (F(4, N) ═ 2.083, *P* ═ 0.121, partial η^2^ ═ 0.294). These findings suggest that while BV/TV values significantly changed over time, this change occurred uniformly across groups, indicating no differential rates of change.

The linear time effect was also statistically significant for the Tb.Th. parameter (F(1, df) ═ 41.354, *P* < 0.001, partial η^2^ ═ 0.674), reflecting a strong influence of time on the measurements. In contrast, the interaction between time and group was significant for the linear contrast (F(4, df) ═ 7.696, *P* ═ 0.001, partial η^2^ ═ 0.606), indicating that changes in Tb.Th. varied among groups, with some displaying significant increases while others exhibited more limited changes. Similarly, the Tb.Sp. parameter demonstrated significant differences over time (F(1, df) ═ 13.894, *P* ═ 0.001, partial η^2^ ═ 0.410), reinforcing the notion that time alone substantially impacted the measurements. The interaction between time and group was also pronounced and significant (F(4, df) ═ 51.082, *P* < 0.0001, partial η^2^ ═ 0.911), highlighting significant divergences in Tb.Sp. changes across groups (Table 1).

The absence of a significant time × group interaction for BV/TV negated the need for Wilcoxon tests or other analyses, indicating that the groups did not exhibit differential changes over time. Multiple comparison corrections were applied to *P* values for comparisons of Tb.Th. and Tb.Sp. Following Bonferroni correction, no comparisons reached statistical significance (*P* > 0.05). Similarly, evaluations using the Holm correction also failed to achieve significance (*P* > 0.05).

Consequently, values that initially approached significance were ultimately deemed statistically insignificant in the comparisons between weeks 2 and 4 after adjusting for multiple testing.

Both the control and graft groups demonstrated minimal changes in BV/TV over time, with values significantly lower than those observed in experimental treatment groups. Sclerostin-ab markedly increased bone volume fraction (BV/TV), with the most substantial effect noted at the 100% dosage.

Over the four-week period, the Scl-Ab 100% group exhibited the highest BV/TV at 0.730 ± 0.010, showing a statistically significant difference compared to the control group (*P* ═ 0.0001). The Scl-Ab 75% group recorded a BV/TV of 0.648 ± 0.035, and the Scl-Ab 50% group presented a BV/TV of 0.588 ± 0.039, both indicating significant increases in bone volume compared to the control group (*P* ═ 0.002, *P* ═ 0.022, respectively) (Table 2).

**Figure 3.**
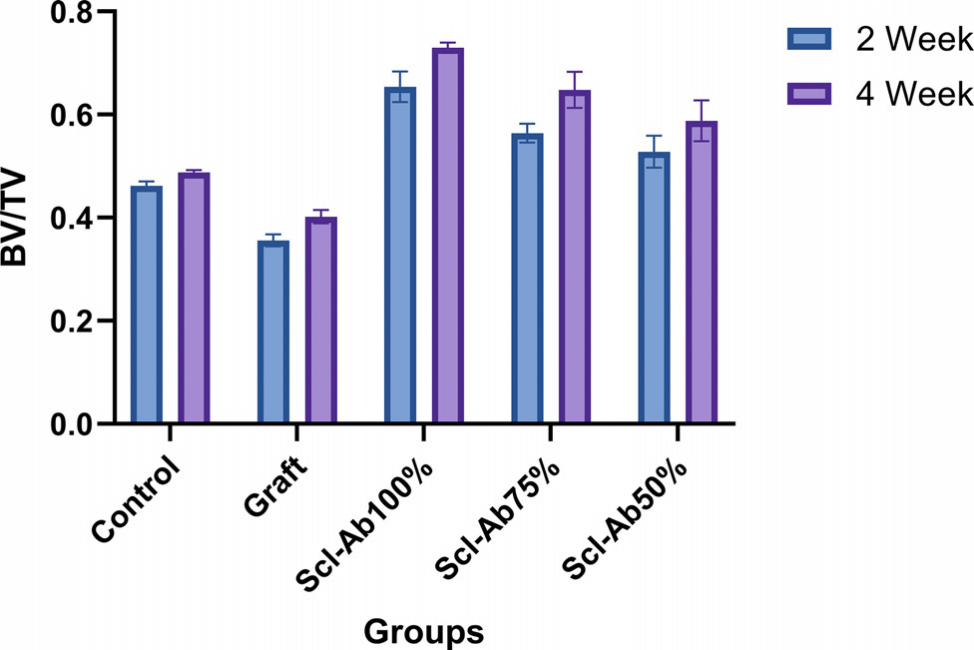
**BV/TV ratios at two time points—week 2 (green bars) and week 4 (burgundy bars)—by treatment group.** This comparison facilitates the assessment of changes in bone volume over time. Images obtained from the cone-beam computed tomography device were analyzed *ex vivo* and measured across axial, sagittal, frontal, and cross-sectional sections using Cybermed On-Demand software, followed by processing with CS_3D imaging software. The region of interest selected from the images enabled volumetric analysis of a specific area within the field of view, focusing on the alveolar bone surrounding the socket of the extracted premolar tooth. In the control group, only tooth extractions were performed, allowing for physiological healing. Graft material was placed post-extraction, with both the right and left sides filled with 50 mg of graft material. The Scl-ab 100% group contained 0.02 mg Scl-ab in 50 mg of graft material, while Scl-ab 75% and Scl-ab 50% groups contained varying ratios of Scl-ab in 50 mg of graft material. A significant difference in BV/TV ratios was observed between the groups at the 2 and 4-week time points. Statistical significance was evaluated using Tukey's and Games-Howell tests. Abbreviations: BV/TV: Bone volume/total volume; Scl-ab: Sclerostin antibody.

Conversely, the graft group recorded a BV/TV of 0.402 ± 0.013 (4 weeks) and 0.356 ± 0.012 (2 weeks), both lower than the control group’s BV/TV of 0.489 ± 0.005 (*P* ═ 0.0001) and 0.459 ± 0.089 (*P* ═ 0.0001), respectively (Figure 3 and Tables 2, 4, and 5).

**Table 1:** Tests of within-subjects effects

**Source**		**Type III sum of squares**	**df**	**Mean square**	**F**	**Sig.**	**Partial eta squared**
BV/TV time	Sphericity assumed	0.043	1	0.043	71.814	0.001	0.782
Time × Groups	Sphericity assumed	0.005	4	0.001	2.083	0.121	0.294
Error (time)	Sphericity assumed	0.012	20	0.001			
Tb.Th. time	Sphericity assumed	0.002	1	0.002	41.354	0.001	0.674
Time × Groups	Sphericity assumed	0.001	4	0.000	7.696	0.001	0.606
Error (time)	Sphericity assumed	0.001	20	3.807E-5			
Tb.Sp. time	Sphericity assumed	0.000	1	0.000	13.894	0.001	0.410
Time × Groups	Sphericity assumed	0.003	4	0.001	51.082	0.001	0.911
Error (time)	Sphericity assumed	0.000	20	1.440E-5			

Abbreviations: BV/TV: Bone volume/total volume; Tb.Th.: Trabecular thickness; Tb.Sp.: Trabecular separation; df: Degrees of freedom.

**Table 2:** CBCT bone morphometry by group at 2 and 4 weeks (*n* ═ 5): BV/TV, Tb.Th., Tb.Sp. (mean ± SD; 95% CI)

**Parameters *n* ═ 5**		**Mean**	**Std. deviation**	**95% CI for mean**	
				**Lower bound**	**Upper bound**
BV/TV	Control	0.4597	0.00895	0.4486	0.4708
2 week	Graft	0.3563	0.01157	0.3420	0.3707
	Scl-ab 100%	0.6540	0.02966	0.6172	0.6908
	Scl-ab 75%	0.5640	0.01817	0.5414	0.5866
	Scl-ab 50%	0.5280	0.03114	0.4893	0.5667
BV/TV	Control	0.4884	0.00477	0.4825	0.4943
4 week	Graft	0.4020	0.01304	0.3858	0.4182
	Scl-ab 100%	0.7300	0.01000	0.7176	0.7424
	Scl-ab 75%	0.6480	0.03493	0.6046	0.6914
	Scl-ab 50%	0.5880	0.03962	0.5388	0.6372
Tb.Th.	Control	0.1478	0.00217	0.1451	0.1505
2 week	Graft	0.1207	0.00894	0.1096	0.1318
	Scl-ab 100%	0.1756	0.00351	0.1712	0.1800
	Scl-ab 75%	0.1680	0.00837	0.1576	0.1784
	Scl-ab 50%	0.1580	0.00837	0.1476	0.1684
Tb.Th.	Control	0.1632	0.00045	0.1626	0.1638
4 week	Graft	0.1407	0.00579	0.1335	0.1479
	Scl-ab 100%	0.1964	0.00410	0.1913	0.2015
	Scl-ab 75%	0.1720	0.00837	0.1616	0.1824
	Scl-ab 50%	0.1540	0.00894	0.1429	0.1651
Tb.Sp.	Control	0.0699	0.00028	0.0696	0.0703
2 week	Graft	0.0439	0.00415	0.0387	0.0490
	Scl-ab 100%	0.0696	0.00546	0.0628	0.0764
	Scl-ab 75%	0.0600	0.00707	0.0512	0.0688
	Scl-ab 50%	0.0460	0.00548	0.0392	0.0528
Tb.Sp.	Control	0.0840	0.00001	0.0840	0.0840
4 week	Graft	0.0500	0.00001	0.0500	0.0500
	Scl-ab 100%	0.0704	0.00550	0.0636	0.0772
	Scl-ab 75%	0.0300	0.00001	0.0300	0.0300
	Scl-ab 50%	0.0350	0.00001	0.0350	0.0350

CBCT-based volumetric measures from a standardized ROI around premolar sockets (24.63 mm^2^; alveolar crest to apex) to assess treatment effects on bone volume and microarchitecture. Outcomes: BV/TV (see Figure 1), Tb.Th., Tb.Sp., at 2 and 4 weeks. ROI metrics were computed via an automated enclosing-convex-polygon workflow (modified BoneJ Density Distribution plugin for ImageJ), incorporating mass/density and concentric-density distributions. Groups (*n* ═ 5 per group): G1 Control (physiologic healing); G2 Graft (50 mg; right side = 2 weeks, left side = 4 weeks filled); G3 Scl-ab 100% (0.02 mg Scl-ab + 50 mg graft); G4 Scl-ab 75%; G5 Scl-ab 50% (both in 50 mg graft). Values are mean ± SD with 95% CI (*n* ═ number of animals). Abbreviations: CBCT: Cone-beam computed tomography; ROI: Region of interest; BV/TV: Bone volume/total volume; Tb.Th.: Trabecular thickness; Tb.Sp.: Trabecular separation; *n*: Number of animals; SD: Standard deviation; CI: Confidence interval; Scl-Ab: Sclerostin antibody.

**Table 3:** Between-group effects (Type III sums of squares) for BV/TV, Tb.Th., and Tb.Sp.

**Source**	**Type III sum of squares**	**df**	**Mean square**	**F**	**Sig.**	**Partial eta squared**
*BV/TV*						
Intercept	14.680	1	14.680	29831.770	0.0001	0.999
Groups	0.580	4	0.145	294.578	0.0001	0.983
Error	0.010	20	0.000			
*Tb.Th.*						
Intercept	1.274	1	1.274	25788.769	0.0001	0.999
Groups	0.017	4	0.004	84.457	0.0001	0.944
Error	0.001	20	4.941E-5			
*Tb.Sp.*						
Intercept	0.156	1	0.156	9139.569	0.0001	0.998
Groups	0.011	4	0.003	157.851	0.0001	0.969
Error	0.000	20	1.708E-5			

The findings show that the group factor has a powerful effect on all BV/TV, Tb.Th., and Tb.Sp. parameters. All differences are highly statistically significant. The variance analysis between groups was conducted, and the main effect of the independent variable (group) on BV/TV, Tb.Th., and Tb.Sp. was evaluated. Abbreviations: BV/TV: Bone volume/total volume; Tb.Th.: Trabecular thickness; Tb.Sp.: Trabecular separation; df: Degrees of freedom.

**Table 4:** Multiple comparisons of BV/TV at 2 weeks

**(I) Groups BV/TV**	**(J) Groups BV/TV**	**Mean difference (I–J)**	**Std. error**	**Sig.**	**95% confidence interval**	
					**Lower bound**	**Upper bound**
Control	Graft	0.10336^*^	0.01384	0.0001	0.0620	0.1448
	Scl-ab 100%	−0.19430^*^	0.01384	0.0001	−0.2357	−0.1529
	Scl-ab 75%	−0.10430^*^	0.01384	0.0001	−0.1457	−0.0629
	Scl-ab 50%	−0.06830^*^	0.01384	0.0010	−0.1097	−0.0269
Graft	Control	−0.10336^*^	0.01384	0.0001	−0.1448	−0.0620
	Scl-ab 100%	−0.29766^*^	0.01384	0.0001	−0.3391	−0.2563
	Scl-ab 75%	−0.20766^*^	0.01384	0.0001	−0.2491	−0.1663
	Scl-ab 50%	−0.17166^*^	0.01384	0.0001	−0.2131	−0.1303
Scl-ab 100%	Control	0.19430^*^	0.01384	0.0001	0.1529	0.2357
	Graft	0.29766^*^	0.01384	0.0001	0.2563	0.3391
	Scl-ab 75%	0.09000^*^	0.01384	0.0001	0.0486	0.1314
	Scl-ab 50%	0.12600^*^	0.01384	0.0001	0.0846	0.1674
Scl-ab 75%	Control	0.10430^*^	0.01384	0.0001	0.0629	0.1457
	Graft	0.20766^*^	0.01384	0.0001	0.1663	0.2491
	Scl-ab 100%	−0.09000^*^	0.01384	0.0001	−0.1314	−0.0486
	Scl-ab 50%	0.03600	0.01384	0.1080	−0.0054	0.0774
Scl-ab 50%	Control	0.06830^*^	0.01384	0.0010	0.0269	0.1097
	Graft	0.17166^*^	0.01384	0.0001	0.1303	0.2131
	Scl-ab 100%	−0.12600^*^	0.01384	0.0001	−0.1674	−0.0846
	Scl-ab 75%	−0.03600	0.01384	0.1080	−0.0774	0.0054

*The mean difference is significant at the 0.05 level (Tukey-HSD). Abbreviations: BV/TV: Bone volume/total volume; Scl-ab: Sclerostin antibody.

**Table 5:** Multiple comparisons of BV/TV at 4 weeks

**(I) Groups BV/TV**	**(J) Groups BV/TV**	**Mean difference (I-J)**	**Std. error**	**Sig.**	**95% confidence interval**	
					**Lower bound**	**Upper bound**
Control	Graft	0.08640^*^	0.00621	0.0001	0.0616	0.1112
	Scl-ab 100%	−0.24160^*^	0.00496	0.0001	−0.2605	−0.2227
	Scl-ab 75%	−0.15960^*^	0.01577	0.0020	−0.2284	−0.0908
	Scl-ab 50%	−0.09960^*^	0.01785	0.0220	−0.1778	−0.0214
Graft	Control	−0.08640^*^	0.00621	0.0001	−0.1112	−0.0616
	Scl-ab 100%	−0.32800^*^	0.00735	0.0001	−0.3538	−0.3022
	Scl-ab 75%	−0.24600^*^	0.01667	0.0001	−0.3124	−0.1796
	Scl-ab 50%	−0.18600^*^	0.01865	0.0010	−0.2617	−0.1103
Scl-ab 100%	Control	0.24160^*^	0.00496	0.0001	0.2227	0.2605
	Graft	0.32800^*^	0.00735	0.0001	0.3022	0.3538
	Scl-ab 75%	0.08200^*^	0.01625	0.0240	0.0148	0.1492
	Scl-ab 50%	0.14200^*^	0.01828	0.0050	0.0653	0.2187
Scl-ab 75%	Control	0.15960^*^	0.01577	0.0020	0.0908	0.2284
	Graft	0.24600^*^	0.01667	0.0001	0.1796	0.3124
	Scl-ab 100%	−0.08200^*^	0.01625	0.0240	−0.1492	−0.0148
	Scl-ab 50%	0.06000	0.02362	0.1750	−0.0219	0.1419
Scl-ab 50%	Control	0.09960^*^	0.01785	0.0220	0.0214	0.1778
	Graft	0.18600^*^	0.01865	0.0010	0.1103	0.2617
	Scl-ab 100%	−0.14200^*^	0.01828	0.0050	−0.2187	−0.0653
	Scl-ab 75%	−0.06000	0.02362	0.1750	−0.1419	0.0219

*The mean difference is significant at the 0.05 level (Game s-Howell test). Abbreviations: BV/TV: Bone volume/total volume; Scl-A b: Sclerostin antibody.

Between-group analysis revealed a significant intercept effect for the BV/TV parameter (F(1, df) ═ 29831.770, *P* < 0.0001, partial η^2^ ═ 0.999). Strong and significant differences were also identified between groups regarding BV/TV values (F(4, df) ═ 294.578, *P* < 0.0001, partial η^2^ ═ 0.983). The high effect size (η^2^p = 0.983) indicates that over 98% of the variance in BV/TV can be attributed to group factors. Similarly, a significant intercept effect was observed for the Tb.Th. value (F(1, df) ═ 25788.769, *P* < 0.0001, partial η^2^ ═ 0.999), revealing substantial differences between groups (F(4, df) ═ 84.457, *P* < 0.0001, partial η^2^ ═ 0.944), with the effect size indicating that approximately 94% of the variance is explained by group factors (Table 3).

Analysis of the Tb.Sp. parameter also revealed a significant intercept effect (F(1, df) ═ 9139.569, *P* < 0.0001, partial η^2^ ═ 0.998), along with statistically significant differences between groups (F(4, df) ═ 157.851, *P* < 0.0001, partial η^2^ ═ 0.969). The group effect was substantial (η^2^p = 0.969), indicating that 97% of the variance in Tb.Sp. values is attributable to group factors. These findings demonstrate that the various groups significantly influenced BV/TV, Tb.Th., and Tb.Sp. values (Table 3).

Data assessment continued with multiple comparisons, revealing significant differences among groups regarding BV/TV, Tb.Th., and Tb.Sp. values at two and four weeks.

During the two-week period, the control and graft groups showed significantly lower BV/TV values compared to the Scl-Ab 100% (*P* ═ 0.0001), Scl-Ab 75% (*P* ═ 0.0001), and Scl-Ab 50% groups (*P* ═ 0.001, *P* ═ 0.0001, respectively). The Scl-Ab 100% group achieved the highest BV/TV ratio and demonstrated a statistically significant increase compared to the control, Scl-Ab 75%, and Scl-Ab 50% groups (*P* ═ 0.0001 for all comparisons). The Scl-Ab 75% group exhibited significantly higher BV/TV values than the control and graft groups (*P* ═ 0.0001 for both) but was significantly lower than the Scl-Ab 100% group (*P* ═ 0.0001). No significant difference was observed for the Scl-Ab 50% group (*P* ═ 0.108). The Scl-Ab 50% group demonstrated a significant increase compared to the graft group (*P* ═ 0.0001) and higher values compared to the control group (*P* ═ 0.001) (Table 4).

After four weeks of treatment, the BV/TV values of the Scl-Ab 75% group were significantly lower than those of the Scl-Ab 100% group (*P* ═ 0.024). The difference between this group and the Scl-Ab 50% group was not statistically significant (*P* ═ 0.175). The Scl-Ab 50% group exhibited significantly higher BV/TV ratios than the graft group (*P* ═ 0.001) and was also significantly higher than the control group (*P* ═ 0.022). However, it had lower values than the Scl-ab 100% group (*P* ═ 0.005), with no significant difference found compared to the Scl-ab 75% group (*P* ═ 0.175) (Table 5).

These dosage results indicate the most significant enhancement over time. Although the 75% and 50% doses of sclerostin-ab also promote increased bone volume, their impacts are less pronounced, underscoring a clear dose-dependent response. Specifically, the Scl-ab 100% group achieved the highest BV/TV ratio, yielding the strongest effect, while the graft group maintained the lowest values, contributing minimally to bone volume.

The second value for the region of interest was the Tb.Th. of the alveolar bone (Tb.Th), which showed a significant enhancement following Scl-ab administration at 2 and 4 weeks. At the 2-week mark, the control group exhibited a Tb.Th of 0.148 ± 0.002 mm, while the graft group displayed a reduced Tb.Th of 0.121 ± 0.009 mm (*P* ═ 0.009). The Scl-ab 100% group achieved the highest Tb.Th of 0.176 ± 0.004 mm compared to both the control and graft groups (*P* ═ 0.0001 for both). Furthermore, the Scl-ab 75% and 50% groups demonstrated increases in Tb.Th, with measurements of 0.168 ± 0.008 mm (significant, *P* ═ 0.022) and 0.158 ± 0.008 mm (not significant, *P* ═ 0.209), respectively, compared to the control group. Both the Scl-ab 75% and 50% groups exhibited significant increases in Tb.Th compared to the graft group (*P* ═ 0.0001 and *P* ═ 0.001, respectively). Although the Scl-ab 75% group had higher Tb.Th values compared to the control and graft groups (*P* ═ 0.022 and *P* ═ 0.0001, respectively), no significant differences were observed between the Scl-ab 75% and Scl-ab 100% or 50% groups (*P* ═ 0.425 and *P* ═ 0.392, respectively). The Scl-ab 50% group also showed a significant increase compared to the graft group (*P* ═ 0.001), but differences were not statistically significant when compared to the control and Scl-ab 75% groups. Additionally, the graft group exhibited the lowest Tb.Th values compared to all groups (*P* < 0.05) (Tables 2 and 6).

**Table 6:** Multiple comparisons of Tb.Th. at 2 and 4 weeks

**Dependent variable**	**(I) Groups**	**(J) Groups**	**Mean difference (I-J)**	**Std. error**	**Sig.**	**95% confidence interval**	
						**Lower bound**	**Upper bound**
Tb.Th. (2 week)	Control	Graft	0.02708^*^	0.00411	0.0090	0.0098	0.0444
		Scl-ab 100%	−0.02778^*^	0.00185	0.0001	−0.0345	−0.0211
		Scl-ab 75%	−0.02018^*^	0.00387	0.0220	−0.0363	−0.0040
		Scl-ab 50%	−0.01018	0.00387	0.2090	−0.0263	0.0060
	Graft	Control	−0.02708^*^	0.00411	0.0090	−0.0444	−0.0098
		Scl-ab 100%	−0.05486^*^	0.00429	0.0001	−0.0718	−0.0379
		Scl-ab 75%	−0.04726^*^	0.00548	0.0001	−0.0662	−0.0283
		Scl-ab 50%	−0.03726^*^	0.00548	0.0010	−0.0562	−0.0183
	Scl-ab 100%	Control	0.02778^*^	0.00185	0.0001	0.0211	0.0345
		Graft	0.05486^*^	0.00429	0.0001	0.0379	0.0718
		Scl-ab 75%	0.00760	0.00406	0.4250	−0.0082	0.0234
		Scl-ab 50%	0.01760^*^	0.00406	0.0330	0.0018	0.0334
	Scl-ab 75%	Control	0.02018^*^	0.00387	0.0220	0.0040	0.0363
		Graft	0.04726^*^	0.00548	0.0001	0.0283	0.0662
		Scl-ab 100%	−0.00760	0.00406	0.4250	−0.0234	0.0082
		Scl-ab 50%	0.01000	0.00529	0.3920	−0.0083	0.0283
	Scl-ab 50%	Control	0.01018	0.00387	0.2090	−0.0060	0.0263
		Graft	0.03726^*^	0.00548	0.0010	0.0183	0.0562
		Scl-ab 100%	−0.01760^*^	0.00406	0.0330	−0.0334	−0.0018
		Scl-ab 75%	−0.01000	0.00529	0.3920	−0.0283	0.0083
Tb.Th. (4 week)	Control	Graft	0.02252^*^	0.00260	0.0040	0.0110	0.0340
		Scl-ab 100%	−0.03320^*^	0.00184	0.0001	−0.0413	−0.0251
		Scl-ab 75%	−0.00880	0.00375	0.2910	−0.0254	0.0078
		Scl-ab 50%	0.00920	0.00400	0.3050	−0.0086	0.0270
	Graft	Control	−0.02252^*^	0.00260	0.0040	−0.0340	−0.0110
		Scl-ab 100%	−0.05572^*^	0.00317	0.0001	−0.0670	−0.0445
		Scl-ab 75%	−0.03132^*^	0.00455	0.0010	−0.0475	−0.0151
		Scl-ab 50%	−0.01332	0.00476	0.1360	−0.0305	0.0038
	Scl-ab 100%	Control	0.03320^*^	0.00184	0.0001	0.0251	0.0413
		Graft	0.05572^*^	0.00317	0.0001	0.0445	0.0670
		Scl-ab 75%	0.02440^*^	0.00417	0.0070	0.0086	0.0402
		Scl-ab 50%	0.04240^*^	0.00440	0.0010	0.0255	0.0593
	Scl-ab 75%	Control	0.00880	0.00375	0.2910	−0.0078	0.0254
		Graft	0.03132^*^	0.00455	0.0010	0.0151	0.0475
		Scl-ab 100%	−0.02440^*^	0.00417	0.0070	−0.0402	−0.0086
		Scl-ab 50%	0.01800	0.00548	0.0603	−0.0009	0.0369
	Scl-ab 50%	Control	−0.00920	0.00400	0.3050	−0.0270	0.0086
		Graft	0.01332	0.00476	0.1360	−0.0038	0.0305
		Scl-ab 100%	−0.04240^*^	0.00440	0.0001	−0.0593	−0.0255
		Scl-ab 75%	−0.01800	0.00548	0.0630	−0.0369	0.0009

Multiple comparisons were performed using the Games–Howell test. Abbreviations: Tb.Th.: Trabecular thickness; Scl-ab: Sclerostin antibody; Std.Error: Standard error.

After 4 weeks of treatment, the Scl-ab 100% group displayed the highest Tb.Th of 0.196 ± 0.004 mm compared to the control and graft groups (*P* ═ 0.0001 for both). The Scl-ab 75% group did not differ significantly from the control group (0.172 ± 0.008 mm, *P* ═ 0.291) but was significantly different from the graft group (*P* ═ 0.001). The Scl-ab 50% group showed values (0.154 ± 0.009 mm) that were not significantly different from the control group (*P* ═ 0.305) or the graft group (*P* ═ 0.136), although it recorded significantly lower Tb.Th values compared to the Scl-ab 100% group (*P* ═ 0.001). The graft group again exhibited the lowest Tb.Th values, which were significantly lower than those of the control group (*P* ═ 0.004) and not different from the Scl-ab 50% group (*P* ═ 0.136) (Tables 2 and 6).

At the 2-week mark, only the comparison between Scl-ab 100% and Scl-ab 715% for Tb.Th was not significantly different (*P* ═ 0.425). However, after 4 weeks, all doses were statistically significantly different (*P* < 0.05, Table 6).

These findings clearly indicate that the graft group exhibited the lowest Tb.Th values at both time points, while the Scl-ab 100% group achieved the highest values (Table 2).

Furthermore, the effect of Scl-ab 100% application became more pronounced over time, while the Scl-ab 75% and 50% groups exhibited values closer to the control level. These results suggest that Scl-ab application has a dose-dependent effect, with the 100% application playing a particularly strong role in enhancing Tb.Th.

Regarding the multiple comparisons of Tb.Sp. values at the 2-week mark, the control group demonstrated a Tb.Sp. of 0.0699 ± 0.0003 mm, while the graft group showed a Tb.Sp. of 0.0439 ± 0.0042 mm (*P* ═ 0.0001). The Scl-ab 100% group exhibited a similar Tb.Sp. of 0.0696 ± 0.0055 mm compared to the control group (*P* > 0.05). However, the Scl-ab 75% group showed a significant reduction, measuring 0.0600 ± 0.0070 mm compared to the control group (*P* ═ 0.039) but was higher than the graft group (*P* ═ 0.001). The Scl-ab 50% group recorded a Tb.Sp. of 0.046 ± 0.0055 mm, significantly lower than the control group (*P* ═ 0.0001) but similar to the graft group (*P* ═ 0.962) (Tables 2 and 7).

**Table 7:** Multiple comparisons of Tb.Sp. at 2 weeks

**(I) Groups**	**(J) Groups**	**Mean difference (I-J)**	**Std. error**	**Sig.**	**95% confidence interval**	
					**Lower bound**	**Upper bound**
Control	Graft	0.02604^*^	0.00319	0.0001	0.0165	0.0356
	Scl-ab 100%	0.00032	0.00319	1.0000	−0.0092	0.0099
	Scl-ab 75%	0.00992^*^	0.00319	0.0390	0.0004	0.0195
	Scl-ab 50%	0.02392^*^	0.00319	0.0001	0.0144	0.0335
Graft	Control	−0.02604^*^	0.00319	0.0001	−0.0356	−0.0165
	Scl-ab 100%	−0.02572^*^	0.00319	0.0001	−0.0353	−0.0162
	Scl-ab 75%	−0.01612^*^	0.00319	0.0010	−0.0257	−0.0066
	Scl-ab 50%	−0.00212	0.00319	0.9620	−0.0117	0.0074
Scl-ab 100%	Control	−0.00032	0.00319	1.000	−0.0099	0.0092
	Graft	0.02572^*^	0.00319	0.0001	0.0162	0.0353
	Scl-ab 75%	0.00960^*^	0.00319	0.0480	0.0001	0.0191
	Scl-ab 50%	0.02360^*^	0.00319	0.0001	0.0141	0.0331
Scl-ab 75%	Control	−0.00992^*^	0.00319	0.0390	−0.0195	−0.0004
	Graft	0.01612^*^	0.00319	0.0010	0.0066	0.0257
	Scl-ab 100%	−0.00960^*^	0.00319	0.0480	−0.0191	−0.0001
	Scl-ab 50%	0.01400^*^	0.00319	0.0020	0.0045	0.0235
Scl-ab 50%	Control	−0.02392^*^	0.00319	0.0001	−0.0335	−0.0144
	Graft	0.00212	0.00319	0.9620	−0.0074	0.0117
	Scl-ab 100%	−0.02360^*^	0.00319	0.0001	−0.0331	−0.0141
	Scl-ab 75%	−0.01400^*^	0.00319	0.0020	−0.0235	−0.0045

*The mean difference is significant at the 0.05 level (Tukey HSD). Abbreviations: Tb.Sp.: Trabecular separation; Scl-ab: Sclerostin antibody; Std. Error: Standard error.

The evaluation of Tb.Sp. indicated that the control and Scl-ab 100% groups exhibited the highest Tb.Th. of the marrow cavities. In contrast, the graft and Scl-ab 50% groups displayed the weakest trabecular structure, while the Scl-ab 75% group showed Tb.Sp. results significantly closer to those of the control and Scl-ab 100% groups.

During the 4-week evaluation, the control group (0.0840 ± 0.0001 mm) had significantly higher Tb.Sp. values compared to the graft group (*P* ═ 0.0001), Scl-ab 100% (*P* ═ 0.024), Scl-ab 75% (*P* ═ 0.0001), and Scl-ab 50% (*P* ═ 0.0001). The graft group (0.0500 ± 0.0001 mm) exhibited higher Tb.Sp. values compared to Scl-ab 75% (*P* ═ 0.0001) and 50% (*P* ═ 0.0001), but was significantly lower than the Scl-ab 100% (*P* ═ 0.005) and control group (*P* ═ 0.0001). The Scl-ab 100% group (0.0704 ± 0.0055 mm) had significantly higher Tb.Sp. values than the graft (*P* ═ 0.005), Scl-ab 75% (*P* ═ 0.0001), and Scl-ab 50% (*P* ═ 0.001) groups, but were significantly lower than the control group (*P* ═ 0.24). The Scl-ab 75% group exhibited significantly lower Tb.Sp. values compared to the control, graft, and Scl-ab 100% groups (*P* ═ 0.0001 for all), showing similarity to the Scl-ab 50% group. Similarly, the Scl-ab 50% group demonstrated significantly lower Tb.Sp. values compared to the control, graft, and Scl-ab 100% groups (*P* < 0.001 for all) (Table 8).

**Table 8:** Multiple comparisons of Tb.Sp. at 4 weeks

**(I) Groups**	**(J) Groups**	**Mean difference (I-J)**	**Std. error**	**Sig.**	**95% confidence interval**	
					**Lower bound**	**Upper bound**
Control	Graft	0.03400^*^	0.00000	0.0001	0.0340	0.0340
	Scl-ab 100%	0.01360^*^	0.00246	0.0240	0.0027	0.0245
	Scl-ab 75%	0.05400^*^	0.00000	0.0001	0.0540	0.0540
	Scl-ab 50%	0.04900^*^	0.00000	0.0001	0.0490	0.0490
Graft	Control	−0.03400^*^	0.00000	0.0001	−0.0340	−0.0340
	Scl-ab 100%	−0.02040^*^	0.00246	0.0050	−0.0313	−0.0095
	Scl-ab 75%	0.02000^*^	0.00000	0.0001	0.0200	0.0200
	Scl-ab 50%	0.01500^*^	0.00000	0.0001	0.0150	0.0150
Scl-ab 100%	Control	−0.01360^*^	0.00246	0.0240	−0.0245	−0.0027
	Graft	0.02040^*^	0.00246	0.0050	0.0095	0.0313
	Scl-ab 75%	0.04040^*^	0.00246	0.0001	0.0295	0.0513
	Scl-ab 50%	0.03540^*^	0.00246	0.0010	0.0245	0.0463
Scl-ab 75%	Control	−0.05400^*^	0.00000	0.0001	−0.0540	−0.0540
	Graft	−0.02000^*^	0.00000	0.0001	−0.0200	−0.0200
	Scl-ab 100%	−0.04040^*^	0.00246	0.0001	−0.0513	−0.0295
	Scl-ab 50%	−0.00500	0.00000	−	−0.0050	−0.0050
Scl-ab 50%	Control	−0.04900^*^	0.00000	0.0001	−0.0490	−0.0490
	Graft	−0.01500^*^	0.00000	0.0001	−0.0150	−0.0150
	Scl-ab 100%	−0.03540^*^	0.00246	0.0010	−0.0463	−0.0245
	Scl-ab 75%	0.00500	0.00000	−	0.0050	0.0050

*The mean difference is significant at the 0.05 level (Games–Howell). Abbreviations: Tb.Sp.: Trabecular separation; Scl-ab: Sclerostin antibody; Std. Error: Standard error.

**Figure 4.**
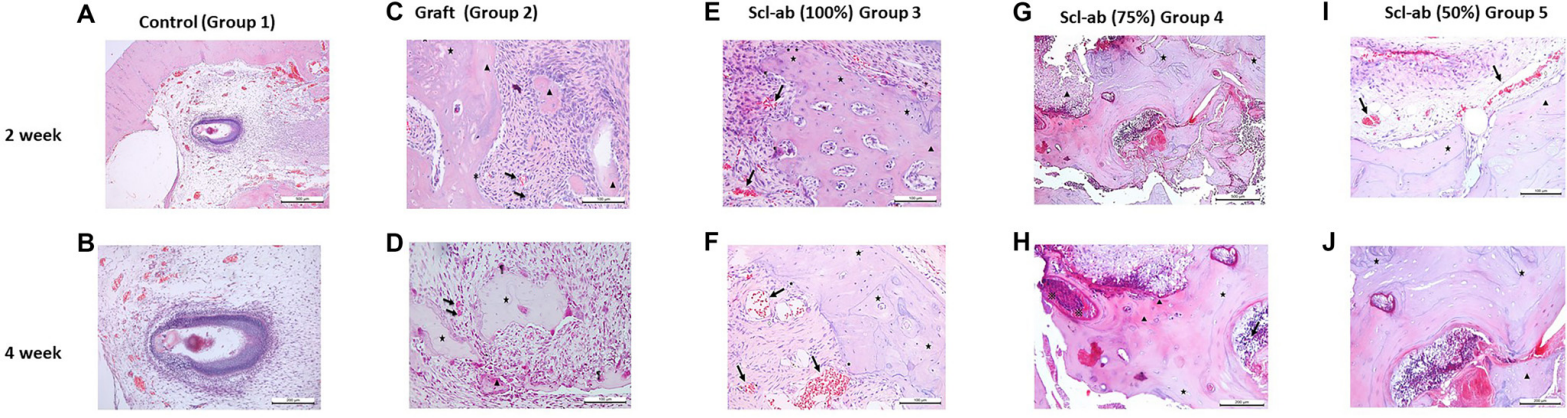
**Histological comparison of socket healing in control, graft, and Scl-Ab groups.** The sections were stained with hematoxylin and eosin (H&E). (A,B) The Control Group (Group 1), which underwent only tooth extraction, demonstrated physiological healing after 2 weeks (A) and 4 weeks (B). (C) In the graft group (Group 2) at 2 weeks, various stages of bone formation are visible: early stage (▴) and advanced stage (★). An increase in capillarization is indicated by the arrow. Osteoblasts were identified on the outer surface of the newly formed bone trabeculae (*). Scale bar: 100 µm. (D) In the graft group at 4 weeks, capillary formations were observed to be less abundant than in the group that received the graft after 2 weeks (arrow). Bone trabeculae formation was evident at different stages (early stage ▴, advanced stage ★), along with the presence of multinucleated giant cells. Scale bar: 100 µm. In all Scl-Ab groups at 2 weeks, (E) Scl-Ab (100%) Group 3, (G) Scl-Ab (75%) Group 4, and (I) Scl-Ab (50%) Group 5 show areas with vascularization (arrow), osteoblasts (*), and newly formed bone trabeculae at varying stages of development (early stage ▴, advanced stage ★). These findings facilitated the evaluation of graft monitoring in specific areas (⋇). Scale bars: E, 100 µm; G, 500 µm; I, 100 µm. In the Scl-Ab groups at 4 weeks, (F), (H), and (J) reveal areas with vascularization (arrow) and newly formed bone trabeculae (early stage ▴, advanced stage ★). Scale bars: F, 100 µm; H, 200 µm; J, 100 µm. This staining method enabled visual assessment of cellular and tissue structures, allowing for an evaluation of the healing processes and tissue organization in the samples. Group 2, which involved graft application, yielded notable results; notably, after four weeks, the level of capillarization was lower in the graft group compared to the group that received the graft after 2 weeks. Abbreviation: Scl-ab: Sclerostin antibody.

These results indicate that the control group exhibited the weakest trabecular structure, characterized by the highest Tb.Sp. values over the four-week period, while the Scl-ab 75% and 50% groups displayed the tightest trabecular structure with the lowest Tb.Sp. values. Furthermore, the Scl-ab 100% application in the early period (week 2) had comparable trabecular spaces to the control group, but this effect diminished in the later period (week 4), as the positive effects of Scl-ab 75% and 50% applications became more pronounced over time (Tables 7 and 8).

### Histological results

The histological observations are illustrated in Figure 4, which serves as a foundation for identifying potential changes when comparing the control group with the graft and experimental groups. This comparison was essential for determining deviations in tissue response and healing patterns, thereby providing insights into the effects of various interventions on tissue regeneration and bone formation. To comprehensively assess the outcomes, we conducted a detailed histological analysis to identify differences among the groups. Hematoxylin and eosin (H&E) staining was employed as the standard technique, as outlined in the materials and methods section under histological analysis.

Both groups that received the graft at 2 and 4 weeks exhibited lower levels of vascularization and regular bone formation compared to the Scl-ab groups. However, after four weeks, the graft group demonstrated more advanced bone growth than the group assessed at two weeks, as shown in Figure 4C and 4D. In Group 3, which received the graft and 100% Scl-ab, significantly increased vascularization was observed in both the 2- and 4-week groups. Unlike the graft group, new capillary formation did not regress in these Scl-ab groups. Furthermore, the number of inflammatory cells increased significantly. The newly formed bone tissue appeared most mature and exhibited the highest amount of newly formed bone trabeculae in the 100% Scl-ab groups at both the 2- and 4-week marks, as supported by the data in Figure 4E.

Additionally, capillarization was more pronounced in the Scl-ab 100% group at the 4-week interval, as indicated in Figure 4F. A well-developed vascular network was observed in Group 4, which received a combination of graft and 75% Scl-ab, with capillarization notably higher than that observed at the 2-week interval (Figure 4G and 4H). In Group 5, which received graft and 50% Scl-ab, histological analysis revealed favorable outcomes concerning connective tissue density and bone formation.

## Discussion

Our group conducted a comprehensive study to assess the effects of locally administering Scl-ab on bone formation following tooth extraction and grafting. This research’s strength lies in its focus on the therapeutic potential of Scl-ab to enhance bone regeneration and improve bone quality in extraction sockets. While various models of bone loss or injury, such as femoral or alveolar defect models, were preclinically investigated, these models are not directly translatable to the clinical context of tooth extraction. The jawbones exhibit distinct characteristics due to differing origins of bone tissues [24, 25].

The primary objective of our research was to investigate the effect of Scl-ab on bone formation using graft materials in tooth sockets. After completing CBCT analysis of the mandibles, we identified regions of interest for volumetric analysis, where enhanced bone regeneration in the mandibular region was observed.

Our analysis indicated that tooth extraction sockets treated with varying ratios of Scl-ab and graft material showed a statistically significant increase in the mean mandibular BV/TV ratio compared to the control and graft groups (Figure 3 and Table 2), influenced by both time and dosage. Although bone volume improved over time, the most remarkable increase was noted in the 100% Scl-ab group. The administration of different doses of Scl-ab also significantly increased the Tb.Th. of alveolar bone, which is the average thickness of all bone voxels [26], a vital indicator of bone quality, microstructure, and formation in a clinical setting, compared to the control (*P* < 0.001) and graft (*P* < 0.001) groups, with histological analysis supporting these findings. However, no significant differences were observed between the 4-week treatment groups of Scl-ab 50% and the control and graft groups, nor between Scl-ab 75% and the control and Scl-ab 50% groups (*P* > 0.05).

This increase was consistent across the 2-week and 4-week treatment periods, highlighting the positive effect of Scl-ab on mandibular bone formation. Results suggest that Scl-ab contributes to overall bone volume and enhances the structural integrity of the trabecular network within the alveolar bone, with efficacy closely linked to the administered dose. After 4 weeks, the Tb.Sp., representing the thickness of the marrow cavities, was lower than that of the control group. Notably, new bone formation increased, and the trabecular structure became denser. Scl-ab treatment is posited to positively affect trabecular bone microarchitecture, reduce intra-bone spaces, and facilitate the integration of new bone tissue, demonstrating the osteogenic effects of sclerostin inhibitors and their potential role in bone regeneration.

Consequently, the observed decrease in Tb.Sp. values indicates that Scl-ab application particularly supports early bone healing and enhances trabecular architecture. This finding underscores the potential use of sclerostin inhibitors as biological agents in regenerative therapies.

Although reliable statistical tests (Welch ANOVA and Brown-Forsythe) demonstrated significant differences between experimental groups for 2 and 4 weeks of BV/TV and Tb.Th, only the 2-week Tb.Sp. data showed significance (*P* < 0.001). Results revealed distinct group-dependent changes in trabecular bone volume and microarchitectural organization; however, robust analysis for 4-week Tb.Sp. data could not be performed due to zero variance in at least one group, limiting reliable statistical estimation. Another limitation was that measurements were conducted by a single experienced operator, which may have reduced variability, although no formal intra-observer ICC analysis was performed.

Researchers have utilized various animal models to gain insights into the intricate workings of the Wnt/β-catenin pathway, including knockout, transgenic, and overexpressing models. These models have proven invaluable in elucidating the complex interactions between this pathway, frizzled receptors, and LRP5/6 on the cell membrane [3, 27]. Furthermore, the Wnt pathway has been implicated in the homeostatic regulation of the periodontal ligament and alveolar bone, mediating force-induced bone modeling during orthodontic tooth movement. Disha-Ibrahimi et al. [28] suggested that during force-induced bone modeling, the downregulation of gene expression levels in the Wnt/beta-catenin signaling pathway directs target genes, with osteocyte maturation related to treatment. In their current study, Turkkahraman et al. investigated the effects of Scl-ab on craniofacial tissues in a healthy and adult-onset mouse model of established type 2 diabetes (T2D), demonstrating severe alveolar bone loss and degeneration of periodontal soft tissues, mirroring the periodontal disease exhibited by T2D patients. Systemic treatment with Scl-ab increased alveolar bone volume, reversed T2D effects on remodeling by increasing osteogenic cells and decreasing osteoclasts, and mitigated bone loss. Remarkably, Scl-ab treatment also exhibited protective effects on periodontal soft tissues, promoting the proliferation of disintegrated periodontal ligament cells, increasing collagen fiber density, preserving extracellular matrix expression, and reducing inflammation [29]. Taut et al. [30] also demonstrated that systemic Scl-ab applied to rats with experimental periodontitis improved alveolar bone mass. In another study, Yao et al. [31] created alveolar bone defects in rats and investigated the efficacy of systemically vs low-dose locally delivered Scl-ab via polylactic-co-glycolic acid microspheres (MSs), concluding that systemic Scl-ab administration improved bone regeneration and tended to enhance cementogenesis as measured by histology and microcomputed tomography. However, no studies have evaluated Scl-ab for repairing osseous defects around teeth or determined the efficacy of locally delivered Scl-ab for targeted drug delivery.

Current methods cannot fully reverse or stimulate regrowth of bone loss. By focusing on the local administration of Scl-ab, our study aims to minimize systemic effects, thereby enhancing its potential for clinical application and yielding promising results.

The primary function of bone grafts is to provide mechanical support and stimulate osteogenesis, facilitating bone replacement with the biological properties essential for osseointegration, osteogenesis, osteoconduction, and osteoinduction. Bone grafts serve primarily as a structural framework that fulfills the osteoconductivity criterion of ideal graft characteristics [32]. They are continually evolving to better meet the needs of the patient population, including the demand for custom bone grafts and synthetic grafts that replicate the qualities of autologous grafts. Alveolar bone grafting is a standard procedure in maxillofacial surgery aimed at reconstructing the alveolar ridge to create a stable foundation for dental implants.

A significant challenge in this procedure is achieving successful osseointegration, which requires establishing a direct structural and functional connection between the grafted bone and surrounding tissues. In our graft-applied group, after four weeks, the level of capillarization—defined as the formation of small blood vessels—was lower compared to the two-week mark. This decline in capillary density suggests a potential reduction in angiogenic activity. Vascularization was less pronounced in the graft-only groups, which may have hindered the efficiency of the healing process. Additionally, these groups exhibited less organized bone formation than the Scl-ab groups, indicating that the graft alone may not have provided adequate support for consistent and structured bone regeneration.

Moreover, when comparing the control groups, we observed a marked decline in BV/TV and Tb.Th values, indicating that while graft application alone can facilitate some degree of healing, vascular and bone formation may be enhanced with the incorporation of additional factors such as Scl-ab into the treatment regimen. Sustained capillary formation is indicative of an ongoing angiogenic response, which is crucial for meeting the metabolic demands of the healing tissue. Contemporary bone grafting materials assist in regeneration but cannot fully and efficiently replicate natural bone formation.

Histological examinations revealed that local application of varying doses of Scl-ab to extraction sockets without bone defects stimulated osteogenesis to a more regular and mature level, resulting in a significant increase in vascularization, newly formed bone tissue, and bone trabeculae. The positive effects of Scl-ab on new capillary formation and the increase in osteoblast numbers further validate its potential as an effective treatment for bone repair and regeneration.

In the two-week group, the tissue exhibited a robust combination of our graft material with Scl-ab, demonstrating a significant increase in osteoblast activity. Osteoblasts, the cells responsible for synthesizing new bone matrix, were notably more abundant, indicating an accelerated bone formation process. Furthermore, capillarization was more pronounced in the Scl-ab four-week groups, reflecting a sustained positive effect on new capillary formation. This enhanced capillarization is illustrated in Figure 4F, emphasizing the efficacy of the 100% Scl-ab treatment in promoting both vascular and bone tissue regeneration. Collectively, these findings suggest that the combined application of graft and 100% Scl-ab fosters robust angiogenesis and improves the quality of bone regeneration through increased osteoblast activity and sustained capillary development. In Group 4, which received a combination of graft and 75% Scl-ab, the study demonstrated a well-developed vascular network indicative of effective angiogenesis within the treated area. The degree of capillarization was significantly higher than that observed at the two-week interval, indicating an improvement in vascularization as the healing process progressed.

Additionally, the analysis revealed the presence of lamellation, a characteristic structural feature of mature bone, defined by its organized, layered matrix. However, a reduction in the extent of lamellation was noted, potentially indicating an active phase of bone remodeling. This decrease suggests that the bone is undergoing dynamic changes, likely involving the formation of new bone and resorption of existing bone. Such remodeling activity is essential for adapting to mechanical stresses and maintaining bone integrity during the healing process. These findings are further detailed in Figure 4G and 4H, highlighting the intricate balance between bone formation and remodeling in the combined graft and 75% Scl-ab treatment. In Group 5, histological analysis revealed a favorable outcome regarding connective tissue density and bone formation matrix, indicative of effective graft integration and enhanced osteogenesis. Notably, well-defined trabecular structures within the newly formed bone suggest active bone remodeling processes. The presence of multinucleated giant cells within the tissue supports the occurrence of cellular responses associated with bone resorption and remodeling, suggesting that the application of 50% Scl-ab alongside the graft may enhance bone healing by promoting new bone formation and remodeling existing bone structures.

The number of osteoblasts was counted across all groups, with the highest counts observed at week four in the Scl-ab treated groups, indicating more advanced bone maturation. At two-week assessment, the Scl-ab 100% group demonstrated the highest number of osteoblasts with a count of 14. This was sequentially followed by the Scl-ab 75% group at 11, the Scl-ab 50% group at 10, the graft group at 9, and the control group recording 8 osteoblasts. By week four, a notable reduction in the osteoblast count was observed in the Scl-ab 100% group, decreasing to 12. In contrast, the counts for the remaining groups at the four-week time point were recorded as 13 for Scl-ab 75%, 13 for Scl-ab 50%, 11 for the graft group, and 8 for the control group. This suggests that osteoblasts may have transitioned to osteocytes by week four, coinciding with the critical progression of bone maturation during this period [33, 34].

Treatments targeting sclerostin may hold therapeutic potential for bone-related disorders by promoting bone regeneration and enhancing bone quality in patients [35]. The anabolic response observed in the activity of Scl-ab in our research could represent a significant breakthrough in treating bone-related ailments. Our findings provide valuable insights into the efficacy of local Scl-ab use for bone growth and quality, as well as for shortening the osseointegration period, potentially paving the way for future clinical trials in this domain. Both excess and deficiency of sclerostin significantly impact these factors. Long-term safety and efficacy studies will enhance the validity of these results. We remain optimistic that our research will contribute to the development of more effective treatments for bone loss and other skeletal conditions.

## Conclusion

The local application of a therapeutic protein composition activates the bone healing process by modulating local signaling mechanisms, thereby promoting efficient and natural-like bone healing. The inclusion of a sclerostin antibody stimulates effective bone formation, as demonstrated by histological analysis showing the development of vascularized bone tissue. This composition offers universal applicability when combined with graft materials, ensuring biocompatibility and straightforward application, which enhances its adoption in clinical settings. This innovative approach significantly improves bone volume augmentation and addresses the limitations of traditional bone grafting methods, providing a transformative strategy for dental professionals seeking expedited recovery and superior long-term outcomes.

## Data Availability

The materials described in the manuscript, including all relevant raw data, will be made available to any researcher wishing to use them for non-commercial purposes, provided participant confidentiality is not breached. Please get in touch with the corresponding author (asepicidincel@gmail.com) for further information.
